# Morphological Variations of Coronoid Process in Dry Adult Human Mandibles: A Narrartive Review

**DOI:** 10.31729/jnma.9028

**Published:** 2025-05-31

**Authors:** Santosh Kumar Nirala, Lei Yuan, Syed Mushraf

**Affiliations:** 1 Department of Human Anatomy, Youjiang Medical University for Nationalities Baise, Youjiang District, Guangxi Province, China; 2 Department of Medicine, Chinese Medical Center Baden, Baden, Wurttemberg, Germany; 3 Department of Basic Medical Sciences, Manipal Academy of Higher Education, Manipal, Karnataka, India

**Keywords:** *coronoid process*, *mandible*, *morphological variations*

## Abstract

The coronoid process of the mandible is a significant anatomical structure integral to mastication and mandibular stability. It serves as an attachment site for the masseter and temporalis muscles, crucial for jaw movement and function. Beyond its functional roles, it holds therapeutic value in reconstructive craniomaxillofacial surgeries, where it serves as a versatile graft material for addressing osseous defects such as fractures, alveolar defects, and sinus augmentations.

Anatomically, it exhibits diverse morphological variations, including triangular, hook-shaped, and rounded forms. These variations not only contribute to understanding anatomical differences but also serve as anthropological markers for ethnic characteristics and ancestry in forensic contexts. Radiographic techniques such as orthopantomograms facilitate the identification and analysis of these morphological differences, essential for both clinical assessments and forensic identifications. Gender-specific differences in its morphology highlight the influence of genetic, hormonal, and developmental factors on mandibular structure.

In conclusion, the coronoid process exemplifies a multifaceted anatomical structure with implications spanning clinical practice, anthropological research, and forensic sciences. Understanding its diverse roles and morphological variations enhances surgical outcomes, aids in anthropological studies, and contributes to forensic identifications, emphasizing its enduring relevance in both medical and scientific disciplines.

## INTRODUCTION

Coronoid Process (CP) derives its literal meaning from the term "korone," which in Greek means "like a crown".^1^ It is a narrow, triangular protrusion that borders the mandibular incisurae on the back and extends anteriorly into the ramus. The masseter and temporalis muscles are attached to the lateral surface of CP. CP is essential for both mastication and mandibular stabilization, even though it is rarely brought up when talking about jaw function.^2^ Because of its therapeutic applicability— it can be utilized as a grafting material for the reconstruction of osseous defects including maxillomandibular fractures, alveolar defects, and sinus augmentation. Furthermore, the distinctive characteristics of CP can serve as an anthropological identifier to determine a person's sex, age, gender, and race.^3^

Adult human mandibles have different forms of the coronoid process. Schaffer first defined it as a beak shaped process in 1915,^4^ while Schulz^5^ described it as an S-shaped, low symmetrical, undulating process while others have referred to it as a triangle process. It has also been noted that a double or second coronoid process exists.^6^

## DEVELOPMENT OF MANDIBLE AND ANATOMY OF THE CORONOID PROCESS

During the sixth week of intrauterine development, the mandible starts to ossify.^7^ The Meckel cartilage, which comes from the first pharyngeal arch (or mandibular arch), acts as a foundation for the mandible's formation. Each side's Meckel cartilage, enclosed by a fibrous membrane, develops an ossification center, and these centers eventually merge at the mandibular symphysis via fibrocartilage. At birth, the mandible consists of two separate bones that fuse within the first year. Initially, the gonial angle is about 160 degrees at birth. As teeth develop around age four, the jaw lengthens and widens, reducing the angle to around 140 degrees. By adulthood, the angle further decreases to about 120 degrees.^7^

The convex anterior border of the coronoid process is in continuity with the anterior border of the ramus below, while its concave posterior border delineates the anterior extent of the mandibular notch.^8^ Smooth in texture, the lateral surface provides a site for the insertion of the temporalis and masseter muscles. On the other hand, the medial surface accommodates the insertion of the temporalis muscle, featuring a ridge that originates close to the apex of the process and extends downward and forward towards the inner aspect of the last molar tooth. Situated between the aforementioned ridge and the anterior border is a grooved triangular region, with its upper section serving as an attachment site for the temporalis muscle and the lower part for certain buccinator fibers.

## MORPHOLOGICAL VARIATIONS AND IMAGING

Numerous studies have been conducted on the morphological differences of the coronoid process in adult human mandibles, revealing a variety of forms, including triangular, rounded, and hook-shaped. According to studies, the most popular shape is a triangle, with rounded and hook-shaped versions coming in second and third.^9-13^ These variances are crucial for comprehending anatomical distinctions, but they are also useful indicators for anthropological evaluations and can help distinguish between genders. In addition, there are statistically significant differences in the lengths of the mandibular condyle and coronoid process between boys and females.^11^ These differences arise from either the genetic background or from changes in function that come about as a result of growing. To yet, the majority of these investigations have been conducted with the dry mandibles taken from the corpses of the departed.

Radiographic images are crucial in forensic odontology for revealing imperceptible details that may not be easily observable through standard physical examinations. The utilization of orthopantomograms in maxillofacial radiography serves as a fundamental screening method in dentistry, offering a cost-effective alternative to more advanced imaging techniques such as CT scans, MRIs, and CBCTs. The panoramic view provided by this imaging modality allows for the identification of various anatomical features like the coronoid process, condyle, and sigmoid notch, which can be compared with both antemortem and postmortem records to facilitate the recognition of individuals, whether deceased or alive.

## MORPHOLOGICAL VARIATIONS IN CORONOID PROCESS SHAPES ACROSS STUDIES

As previously indicated, several investigations have revealed differing preponderances of morphologies in the human mandible's coronoid process. Regarding the frequency of the various coronoid process types, different studies have come to different results. Some have shown that triangular processes are the most common, followed by rounded processes and hooked processes.

The coronoid process of the mandible exhibits a diverse range of morphological variations, which have been extensively studied across different populations. Understanding these variations is crucial for clinical practices such as maxillofacial surgery and dental procedures. The following paragraphs critically analyse the findings from various studies on the shapes of the coronoid process. The triangular shape is the most commonly observed form of the coronoid process in the majority of studies. For instance, Purohit^14^ reported an exceptionally high prevalence of triangular shapes, with 72% of the mandibles exhibiting this form. This finding is supported by several other studies, including those by Tanveer A et al. (67%) and Sangal et al (59.2%), which indicate that more than half of the observed coronoid processes are triangular.^15^ Conversely, Kasat et al documented a significantly lower prevalence of triangular shapes at 23.5%, suggesting considerable population-specific variations.^16^ The hook-shaped coronoid process is the second most prevalent variant in many studies. Kasat et al found that 54.5% of the coronoid processes in their sample were hook-shaped, representing the highest recorded prevalence for this shape.^16^ This contrasts sharply with findings by Kiran C et al.^17^ Tapas reported lower hook-shaped prevalence rates of 14.1% and 22% respectively.^17,18^ The variability in hook-shaped coronoid processes highlights the influence of genetic and environmental factors on mandibular morphology. Rounded shapes are generally the least common variant of the coronoid process, with prevalence rates ranging from 3% to 35.3%. Pradhan S et al^19^ observed the highest incidence of rounded shapes at 35.3%, indicating a notable divergence from other studies. For example, Khan TA et al^20^ reported a minimal prevalence of rounded shapes at 3%, suggesting that this form is relatively rare in their study population. Sangal et al^15^ also documented a lower prevalence of 12.8%, reinforcing the notion that rounded coronoid processes are less frequently encountered. In addition to the primary shapes of triangular, hook, and rounded, several studies identified other less common variants. Kasat et al^16^ observed unique shapes such as square (0.5%) and a combined hook and round form (3%). Desai et al^21^ noted truncated (13%), nodular (24%), and assimilated (12%) variants, further emphasizing the morphological diversity of the coronoid process. Gender differences in the morphology of the coronoid process were also noted. Sangal et al^15^ found that the triangular shape predominated in both sexes, with a higher prevalence in males (39.2%) compared to females (20%). Similarly, Gaharwar et al^22^ reported a higher incidence of triangular shapes in both adults (65.62%) and older individuals (60.29%). However, the prevalence of hook and rounded shapes showed less significant gender-based differences.

Overall, the substantial variability in the morphology of the coronoid process across different populations underscores the importance of recognizing these differences in clinical settings. Tailored surgical approaches that consider these morphological variations can enhance patient outcomes in maxillofacial surgery and other related fields. Further research involving larger and more diverse populations is necessary to fully understand the underlying causes and clinical implications of these variations. The existing studies provide a valuable foundation for such investigations, highlighting the complex interplay of genetic, environmental, and developmental factors that shape the anatomy of the coronoid process.

## GENDER DIFFERENCES IN CORONOID PROCESS SHAPES

The study by Kiran C et al^17^ presents a stark contrast between males and females in the prevalence of triangular, hookshaped, and rounded coronoid processes. Males predominantly exhibit the triangular shape (69.4%), whereas females have a lower prevalence (33.3%). Interestingly, the rounded shape is significantly more common in females (46.7%) than in males (19.4%), indicating potential sex-specific developmental influences. The higher percentage of hook-shaped processes in females (20%) compared to males (11.3%) further underscores these differences.

**Table 1:** Shapes Differences

Study	Triangular (%)	Hook Shaped (%)	Rounded (%)	Other Variants (%)
Kiran C et al^17^	57.6	14.1	28.3	-
Desai et al^21^	51	13	24	13 (Truncated) 24 (Nodular) 12 (Assimilated)
Tapas^18^	60	22	18	-
Nirmale et al^23^	65	28	7	-
Prajapati et al^25^	54.17	21.25	24.58	-
Kasat et al^16^	23.5	54.5	18.5	0.5 (Square) 3 (Hook & Round)
Pradhan S et al^19^	46.73	17.93	35.3	-
Sangal et al^15^	59.2	28	12.8	-
Jebaraj S et al^26^	49	27.4	23.6	-
Purohit^14^	72	14	14	-
Singh G et al^27^	40.55	31.67	27.78	-
Gaharwar et al^22^	65.62 (Adults) 60.29 (Old)	25 (Adults) 29.41 (Old)	9.38 (Adults) 10.29 (Old)	_-_

Desai et al^21^ also observed a higher prevalence of triangular shapes in males (39%) compared to females (12%). The study shows that hook-shaped processes are more common in males (11%) than in females (2%), and rounded shapes are more prevalent in males (15%) than in females (9%). This pattern aligns with Kiran C et al^17^ suggesting a consistent trend of more pronounced morphological variability in males.

Pradhan S et al^19^ reported a relatively balanced distribution of triangular shapes between males (45.83%) and females (47.72%). However, females exhibited a higher prevalence of rounded shapes (38.63%) compared to males (32.29%). The hook-shaped coronoid process was more common in males (21.87%) than in females (13.63%). This study highlights the nuanced differences in morphological traits that can vary slightly but significantly between sexes. The findings by Nirmale et al^23^ show a high prevalence of triangular shapes in both males (64.22%) and females (62.5%). The hook-shaped process is equally common in both sexes, with males at 27.52% and females at 27.5%. Rounded shapes are slightly more prevalent in females (10%) compared to males (8.26%). This uniformity across sexes suggests a lesser degree of morphological variation in the studied population. Parveen et al^24^ observed lower overall percentages for all shapes compared to other studies, with males showing a slightly higher prevalence of triangular shapes (34.47%) than females (31.63%). Hook-shaped and rounded processes are relatively rare, with males exhibiting 11.75% and 8.33%, and females 10.22% and 3.60%, respectively. This study emphasizes the variability in morphological traits and the need for population-specific data. Sangal et al^15^ found a higher prevalence of triangular shapes in males (39.2%) compared to females (20%). Both sexes exhibited equal prevalence for hookshaped (15.2%) and rounded shapes (6.8%). This equality in non-triangular shapes contrasts with other studies, suggesting potential genetic or environmental factors influencing these traits. Gaharwar et al^22^ reported that females have a higher prevalence of triangular shapes (36%) compared to males (32%). Males exhibit a higher prevalence of hook-shaped processes (14%) than females (8%), and rounded shapes are more common in females (6%) compared to males (4%). This study highlights sex-specific morphological variations that may have clinical implications.

Overall, these studies demonstrate significant gender differences in the morphological variations of the coronoid process. The prevalence of triangular shapes tends to be higher in males across most studies, whereas females exhibit a greater prevalence of rounded shapes. Hook-shaped processes show less consistent gender differences. These variations underscore the importance of considering sex-specific traits in clinical practice and anatomical studies. The discrepancies among studies also highlight the need for further research to explore the underlying causes of these morphological differences and their potential implications for medical and forensic applications.

## FACTORS INFLUENCING MORPHOLOGICAL VARIATIONS

Numerous factors, including the attachment and function of the temporalis muscle, the habit of unilateral chewing, and hormonal considerations, have been implicated in the variation in the form of the coronoid process. The morphology and development of the coronoid process of the mandible are influenced by a variety of specific factors. Dietary habits, particularly the intake of nutrients like calcium, phosphorus, and vitamin D, play a critical role in bone mineralization and remodeling, thereby impacting the shape and size of the coronoid process. Hormonal influences, such as growth hormone and estrogen, regulate bone growth and development, influencing the morphology of skeletal structures including the coronoid process. Genetic factors contribute to individual variations in mandibular morphology, influencing the shape and projection of the coronoid process based on familial traits and genetic predispositions. Additionally, the activity of the temporalis muscle, which attaches to the coronoid process, is pivotal. The temporalis muscle's strength and usage patterns, influenced by chewing habits and overall craniofacial muscle dynamics, directly impact the development and shape of the coronoid process.

**Table 2:** Gender Differences Observed Between Studies

Study	Gender	Triangular (%)	Hook Shaped (%)	Rounded (%)
Kiran C et al^17^	Male	69.4	11.3	19.4
	Female	33.3	20	46.7
Desai et al^21^	Male	39	11	15
	Female	12	2	9
Praddhan S et al^19^	Male	45.83	21.87	32.29
	Female	47.72	13.63	38.63
Nirmale et al^23^	Male	64.22	27.52	8.26
	Female	62.5	27.5	10
Parveen et al^24^	Male	34.47	11.75	8.33
	Female	31.63	10.22	3.60
Sangal et al^15^	Male	39.2	15.2	6.8
	Female	20	15.2	6.8
Gaharwar et al^22^	Male	32	14	4
	Female	36	8	6

Continuous muscle activity and load-bearing from chewing exert mechanical stress on the coronoid process, potentially influencing its growth and morphology over time. Together, these factors highlight a comprehensive understanding of how dietary, hormonal, genetic, and muscular influences collectively shape the morphology and development of the coronoid process within the mandible.^28-31^

## CLINICAL RELEVANCE

Because the medial aspect of the coronoid process is located so near the distal molar teeth, anatomical differences in the form of the coronoid process may cause the vestibular space to narrow. This can result in impediments, which could limit mouth opening and produce mandibular hypomobility.^32^ The coronoid process, despite its relatively small size, plays pivotal roles in various clinical and anatomical contexts, making it a valuable structure in medical practice and research.

## RECONSTRUCTIVE SURGERY

The coronoid process is highly favoured in reconstructive craniomaxillofacial surgeries due to its unique properties. Its intraoral accessibility allows surgeons to harvest it without causing significant functional impairments or aesthetic disturbances. As a graft material, the coronoid process is utilized in intricate procedures such as orbital floor reconstruction, where its dense cortical bone provides structural support and facilitates tissue regeneration. Moreover, in cases of alveolar defects, the coronoid process can be shaped and integrated to restore dental and facial aesthetics. Its application extends to augmenting paranasal sinuses, where its osteogenic potential aids in maintaining sinus integrity and supporting nasal reconstruction. Additionally, for non-union fractures of the mandible, the coronoid process graft promotes bone healing and stability, contributing to successful surgical outcomes and patient recovery.

## ANTHROPOLOGICAL MARKER

Anthropologically, the morphology of the coronoid process serves as a significant marker for determining ethnic characteristics and ancestry. Studies have shown distinct variations in the shape and size of the coronoid process among different populations, reflecting evolutionary adaptations and genetic influences. Forensic experts utilize these anatomical variations to establish demographic profiles of skeletal remains, assisting in the identification and reconstruction of historical populations. This anthropological application underscores the coronoid process's role in understanding human migration patterns and genetic diversity across diverse cultural landscapes.

## AUTOGENOUS BONE GRAFTS

The coronoid process offers several advantages as a source of autogenous bone grafts compared to alternative donor sites. Its proximity to the surgical site minimizes operative complexities and reduces the risk of postoperative complications associated with distant graft procurement. The dense cortical bone of the coronoid process provides structural support and osteogenic potential essential for successful bone regeneration in reconstructive procedures. While other donor sites like the ilium, rib, and calvaria are viable options, each presents specific challenges such as increased surgical morbidity or limited bone volume. The coronoid process, therefore, stands out as a preferred choice for autogenous bone grafts, particularly in cases requiring precise bone contouring and alignment for optimal functional and aesthetic outcomes.

## CONCLUSION

Beyond its biomechanical roles, the coronoid process holds therapeutic value in reconstructive craniomaxillofacial surgeries, where it serves as a versatile graft material for reconstructing osseous defects such as maxillomandibular fractures, alveolar defects, and sinus augmentations. Its diverse morphological variations, including triangular, hook-shaped, and rounded forms, have been extensively studied across different populations, offering insights into anthropological characteristics and genetic influences. Radiographic imaging techniques, particularly orthopantomograms, play a pivotal role in identifying these variations and their implications for medical and forensic applications. Moreover, gender-specific differences in coronoid process morphology underscore the complex interplay of genetic, hormonal, and environmental factors shaping mandibular anatomy. Overall, the coronoid process exemplifies a multifaceted anatomical structure with implications spanning clinical practice, anthropological research, and forensic sciences, highlighting its enduring relevance in understanding human skeletal diversity and advancing surgical innovations.
